# Differential changes in cyclic adenosine 3′‐5′ monophosphate (cAMP) effectors and major Ca^2+^ handling proteins during diabetic cardiomyopathy

**DOI:** 10.1111/jcmm.17733

**Published:** 2023-03-27

**Authors:** Victoria Chaoul, Rita Hanna, Pia Hachem, Magali Samia El Hayek, Wared Nour‐Eldine, Pamela Abou‐Khalil, Elias Abi‐Ramia, Grégoire Vandecasteele, Aniella Abi‐Gerges

**Affiliations:** ^1^ Gilbert and Rose‐Marie Chagoury School of Medicine Lebanese American University P.O. Box 36 Byblos Lebanon; ^2^ Signaling and Cardiovascular Pathophysiology, UMR‐S1180 Université Paris‐Saclay Orsay 91400 France; ^3^ School of Arts and Sciences, Department of Natural Sciences Lebanese American University Byblos Lebanon

**Keywords:** Ca^2+/^calmodulin‐dependent kinase II, cAMP‐dependent protein kinase, diabetic cardiomyopathy, exchange protein directly activated by cAMP, excitation–contraction coupling, type 1 diabetes

## Abstract

Diabetic cardiomyopathy (DCM) is associated with differential and time‐specific regulation of β‐adrenergic receptors and cardiac cyclic nucleotide phosphodiesterases with consequences for total cyclic adenosine 3′‐5′ monophosphate (cAMP) levels. We aimed to investigate whether these changes are associated with downstream impairments in cAMP and Ca^2+^ signalling in a type 1 diabetes (T1D)‐induced DCM model. T1D was induced in adult male rats by streptozotocin (65 mg/kg) injection. DCM was assessed by cardiac structural and molecular remodelling. We delineated sequential changes affecting the exchange protein (Epac1/2), cAMP‐dependent protein kinase A (PKA) and Ca^2+^/Calmodulin‐dependent kinase II (CaMKII) at 4, 8 and 12 weeks following diabetes, by real‐time quantitative PCR and western blot. Expression of Ca^2+^ ATPase pump (SERCA2a), phospholamban (PLB) and Troponin I (TnI) was also examined. Early upregulation of Epac1 transcripts was noted in diabetic hearts at Week 4, followed by increases in Epac2 mRNA, but not protein levels, at Week 12. Expression of PKA subunits (RI, RIIα and Cα) remained unchanged regardless of the disease stage, whereas CaMKII increased at Week 12 in DCM. Moreover, PLB transcripts were upregulated in diabetic hearts, whereas SERCA2a and TnI gene expression was unchanged irrespective of the disease evolution. PLB phosphorylation at threonine‐17 was increased in DCM, whereas phosphorylation of both PLB at serine‐16 and TnI at serine‐23/24 was unchanged. We show for the first time differential and time‐specific regulations in cardiac cAMP effectors and Ca^2+^ handling proteins, data that may prove useful in proposing new therapeutic approaches in T1D‐induced DCM.

## 1 INTRODUCTION

Cyclic adenosine 3′‐5′ monophosphate (cAMP) is a pivotal regulator of cardiac contractility, relaxation and automaticity. The intracellular levels of this second messenger are finely tuned by their rate of synthesis by adenylyl cyclases (AC), and degradation by cyclic nucleotide phosphodiesterases (PDEs).^1^ In normal myocardium, cAMP elevation, particularly through β‐adrenergic receptor (β‐AR) stimulation, exerts inotropic and lusitropic effects by activating cAMP‐dependent protein kinase (PKA). In its inactive form, PKA holoenzyme is a heterotetramer composed of two regulatory (R) and two catalytic (C) subunits.^2^, ^3^, ^4^ Four types of R subunits (RIα, RIβ, RIIα, and RIIβ) and three C subunits (Cα, Cβ, and Cγ) have been described^5^ with RIα, RIIα and Cα being the major isoforms expressed in the heart^6^ and encoded by distinct genes. Two classes of PKA holoenzymes have been identified (type I and II), which differ in their R subunits (RI and RII)^7^, ^8^ and cellular localization.^9^ Binding of cAMP to PKA‐R subunits causes the dissociation of the tetrameric PKA holoenzyme and thus the release of the free PKA‐C subunits,^10^, ^11^ hence promoting the phosphorylation and activation of key components of the cardiac excitation–contraction coupling (ECC). These include L‐type Ca^2+^ channels (LTCCs) and their constitutive inhibitor Rad,^12^ ryanodine receptors (RyR2) of the sarcoplasmic reticulum (SR), phospholamban [PLB, a constitutive inhibitor of the SR Ca^2+^ pump (SERCA2a)], Troponin I (TnI) and cardiac myosin‐binding protein C (MyBP‐C).^13^ Activation of the main actors of the ECC enhances Ca^2+^ cycling and consequently increases heart rate (HR), contraction amplitude and relaxation.^14^ Along with PKA, Ca^2+^/Calmodulin‐dependent kinase II (CaMKII) contributes to β‐AR regulation of cardiac function^15^, ^16^; LTCCs, RyR2, MyBP‐C and PLB are also substrates for CaMKII.

Emerging evidence supports a relevant role for Epac, a guanine nucleotide‐exchange factor for the small GTPases Rap1 and Rap2,^17^, ^18^, ^19^, ^20^ as a mediator of cAMP signalling in the heart and a regulator of Ca^2+^ signalling/contractility.^21^ Two cardiac Epac isoforms have been identified. Epac1 is the major neonatal isoform whereas Epac2 expression is predominant in adults.^22^ Upon cAMP binding, Epac enhances the phosphorylation of several Ca^2+^ handling proteins including RyR2 and PLB, thereby facilitating Ca^2+^ release from SR and reuptake. Epac also increases cardiac myofilament Ca^2+^ sensitivity through phosphorylation of TnI and MyBP‐C.^23^ These effects have been attributed to the activation of Epac/Rap/Phospholipase C (PLC)/protein kinase C (PKC)/ CaMKII‐mediated signalling.^21^, ^24^, ^25^

Diabetes mellitus (DM) represents a major global health problem^26^ and contributes to the development of diabetic cardiomyopathy (DCM), which finally culminates in heart failure (HF)^27^, ^28^ in the absence of hypertension and structural heart diseases. DCM is characterized by cardiac remodelling, myocardial fibrosis, diastolic and systolic dysfunction.^29^ The underlying pathophysiology of the prolonged process that culminates in DCM and HF is complex. However, it is well accepted that cardiac dysfunction and HF caused by DM are associated with metabolic abnormalities, sympathetic nervous system (SNS) overactivity and Ca^2+^ mishandling.^30^, ^31^ Nevertheless, contrasted results were obtained regarding altered β‐AR‐mediated response and PKA signalling in various animal models of DM.^32^, ^33^ Moreover, there is little information concerning Epac modification in DCM despite its established role in mediating pro‐hypertrophic effects of β‐AR stimulation.^34^ Although disturbances in Ca^2+^ signalling and CaMKII have been investigated in DCM,^30^, ^35^, ^36^ data on actual changes affecting the expression and activity of the proteins involved in Ca^2+^ homeostasis during the evolution of DCM are controversial.^30^, ^31^, ^33^

In a recent study, we characterized the evolution of cardiac structure and function in a rat model of DCM at 4, 8 and 12 weeks after streptozotocin (STZ)–induced type 1 diabetes (T1D).^37^ We showed that sustained hyperglycaemia in diabetic rats was associated with cardiac remodelling including steatosis and fibrosis as well as bradycardia and an early (4 weeks) increase in cardiac systolic function as evaluated by ejection fraction (EF) and fraction shortening (FS) of the left ventricle (LV).^37^ This correlated with upregulation of β_1_‐ARs and total cAMP levels, whereas normalization of cardiac function and cAMP occurred at later time points and correlated with upregulation of some of the major cardiac PDEs.^37^ However, whether these changes were associated with downstream modifications of cAMP effectors and Ca^2+^ handling proteins in DCM was not investigated. Thus, this study was designed to delineate the alterations affecting Epac1/2 isoforms and PKA (RI, RIIα and Cα subunits), as well as the expression of CaMKII and the major actors of the ECC process in the same rat model of STZ‐induced T1D. We show for the first time differential and time‐specific regulations in cardiac Epac1/2, PKA subunits, CaMKII, as well as the impact of these alterations on the phosphorylation status of PLB and TnI. These results may prove useful in proposing new therapeutic approaches in T1D‐induced DCM.

## 2 MATERIALS AND METHODS

### 2.1 Animal model

All experiments were approved by the Animal Care and Use Committee at the Lebanese American University (LAU) and adhered to the Guide for the Care and Use of Laboratory Animals published by the US National Research Council committee.

Eighty‐nine adult male Wistar rats were used in this study. T1D was induced at 5 weeks of age in rats weighing between 80–130 g as previously described.^37^ Briefly, 12 h after fasting, animals received one intraperitoneal injection of streptozotocin (STZ, Sigma‐Aldrich: 65 mg/kg in 0.1 M citrate buffer, pH = 4.5). Age‐matched control rats (CON) were injected with vehicle (0.1 M citrate buffer, pH = 4.5) via the same route. After 72 h, fasting glucose levels were measured in blood droplets withdrawn from the tails of animals, using Accu‐Check® Performa glucometer (Roche). Eighty‐five per cent of the rats injected with STZ exhibited fasting blood glucose (FBG) levels >200 mg/dL in addition to polyuria and polydipsia and were considered diabetic. All experiments were performed at 4, 8 and 12 weeks after STZ or vehicle injection (Figure S1).

### 2.2 Anatomical study

Body weights (BW) were measured under anaesthesia, and the hearts were rapidly excised, rinsed with a fresh cold physiological saline solution, weighed and stored in liquid nitrogen for real‐time PCR for the genes of interest [Atrial natriuretic factor (ANF), Epac1/2, SERCA2a, PLB and TnI] and western blot analysis for Epac2, the main PKA subunits (RI, RIIα and the Cα subunit), CaMKII, SERCA2a, p‐TnI (Ser^23/24^), total TnI, p‐PLB (Ser^16^), p‐PLB (Thr^17^) and total PLB. Lungs, liver and kidneys were also removed from all animals and weighed.

### 2.3 Real‐time quantitative PCR

Total RNA was isolated from the frozen cardiac tissue of all control and diabetic rats using TRIZOL reagent (Ambion; life technologies). RNA (1 μg) was reverse‐transcribed to single‐stranded cDNA using iScript cDNA synthesis kit (Bio‐Rad) as per the manufacturer's instructions. Real‐time qPCR was performed with CFX96 Real‐Time PCR Detection System (Bio‐Rad) using SoAdvancedTM Universal SYBR® Green Supermix (Bio‐Rad) and performed on CFX96 RT‐PCR Detection System (Bio‐Rad) in triplicate. Three independent technical replicates were run for each cardiac sample. The specificity of each primer set was monitored by analysing the dissociation curve. GAPDH was used as a housekeeping gene. Expression of the following genes was analysed: ANF, Epac1/2, SERCA2a, PLB and TnI. Table S1 shows the sequence of forward and reverse primers that were used. The relative expression level of each gene was determined using the comparative cycle threshold (Ct) method (2^−∆∆Ct^) normalized to control GAPDH gene.

### 2.4 Western blot

Frozen hearts from control and diabetic rats at 4, 8 and 12 weeks were homogenized in an ice‐cold buffer containing 150 mM NaCl, 20 mM Hepes (pH 7.4), 2 mM EDTA, 1 mM Phenylmethylsulfonyl fluoride (Sigma‐Aldrich) and supplemented with 10% Glycerol, 0,2% Triton and Complete Protease Inhibitor Tablets from Roche Diagnostics. Protein lysates were kept on ice for 30 min and then centrifuged at 16560 *g* for 15 min at 4°C. The total protein concentration in the supernatant was determined using Nanodrop, and then 20 μg of protein extracts was loaded onto sodium dodecyl sulfate‐polyacrylamide gel electrophoresis gels for separation and transferred to polyvinylidene fluoride membranes (Bio‐Rad). Membranes were blocked with bovine serum albumin for 1 h at room temperature and then probed with different primary antibodies at 4°C overnight. Epac2 was detected using a rabbit polyclonal anti‐Epac2 antibody (1:1000; Proteintech 19,103‐1‐AP). PKA subunits (RI, RIIα and Cα) were detected using mouse antibodies (1:500; BD Biosciences). CaMKII was detected with a rabbit anti‐CaMKII antibody (1:1000; Santa Cruz). SERCA2a was detected with a mouse anti‐SERCA2a (1:1000; Santa Cruz). Rabbit anti‐PLB (1:5000; Cell Signaling), rabbit anti‐phospho‐Ser^16^ PLB (1:5000; Badrilla), rabbit anti‐phospho‐Thr^17^ PLB (1:5000; Badrilla), rabbit anti‐TnI (1:1000; Cell Signaling) and rabbit anti‐phospho‐Ser^23^/^24^ TnI (1:1000; Cell Signaling) antibodies were used to detect total PLB, phospho‐Ser^16^ PLB [p‐PLB (Ser^16^)], phospho‐Thr^17^ PLB [p‐PLB (Thr^17^)], total TnI and phospho‐Ser^23^/^24^ TnI [p‐TnI (Ser^23^/^24^)], respectively. Protein loading was monitored using a rabbit anti‐actin (1:1000; Abcam) and a rabbit anti‐calsequestrin (1:2000; Thermo Scientific) antibodies. The membranes were then probed with horseradish peroxidase‐conjugated secondary antibodies: goat pAb to rabbit (Abcam, ab6721) or to mouse (Abcam, ab97040), and the bands were visualized by enhanced chemiluminescence (Clarity Western ECL substrate; Bio‐Rad, 170–5061). Immunoreactive bands were revealed in ChemiDoc Imaging Systems (Bio‐Rad), and densitometric analysis with ImageJ software was used for quantification. For Epac2, PKA subunits (RI, RIIα and Cα), CaMKII, SERCA2a, total TnI and total PLB, each sample was normalized to the loading control (calsequestrin and/or actin): R1 = protein expression/loading control ratio. For p‐TnI (Ser^23/24^), p‐PLB (Ser^16^), p‐PLB (Thr^17^), R2 [phosphorylated protein/loading control ratio] was first quantified for each sample and then normalized to its corresponding total protein/loading control ratio: R2/R1.

### 2.5 Statistical analysis

All quantitative data are presented as mean ± SEM and analysed by Prism (version 8.0). A two‐way anova was performed in order to study the effect of STZ treatment, time and their interaction on the different outcomes. When the interaction was significant, two‐way anova was followed by post hoc Tukey's multiple comparison test. Significance was set to *p* < 0.05.

## 3 RESULTS

### 3.1 Induction and characterization of DCM in adult rats

**FIGURE 1:**
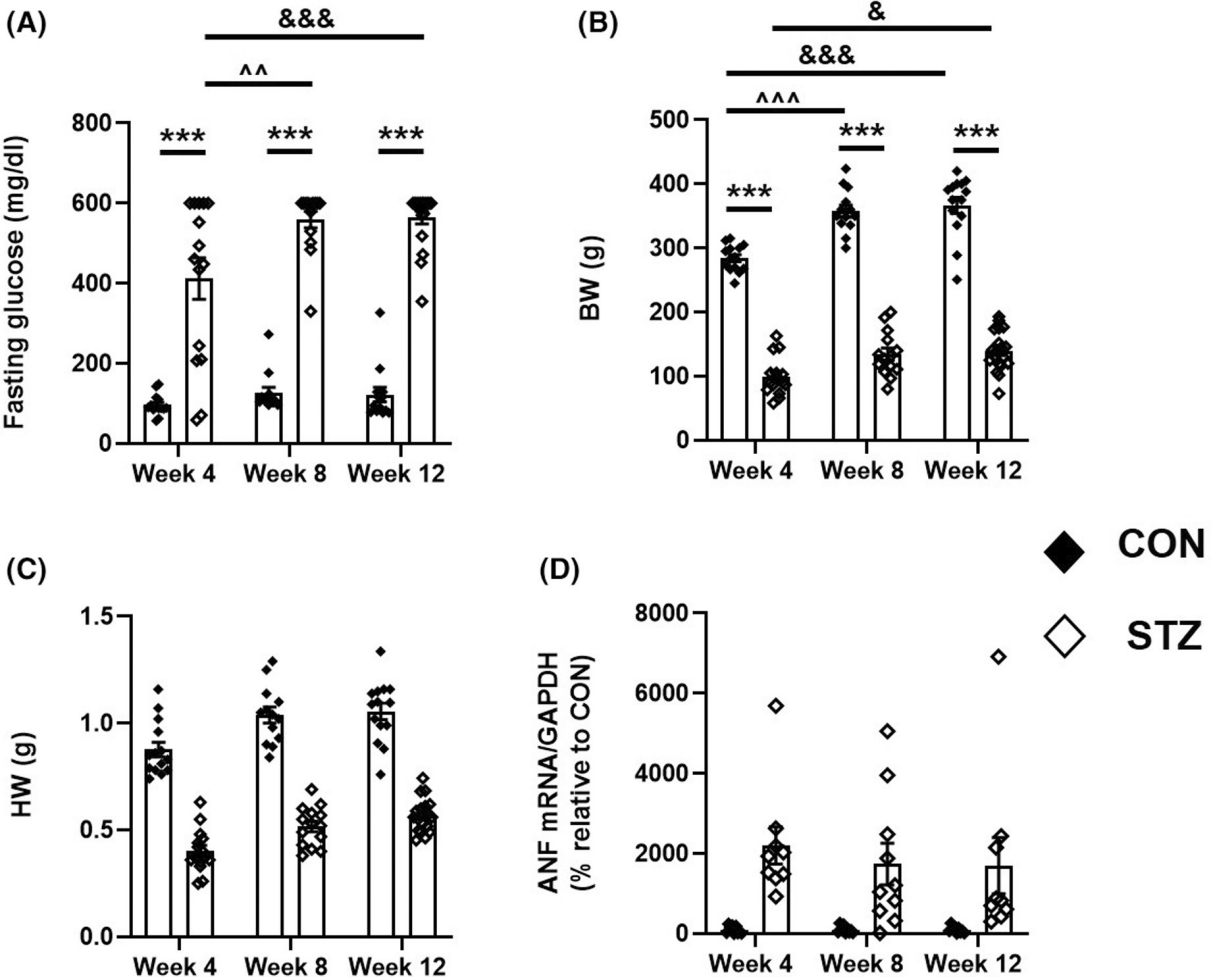
Hyperglycaemia is associated with cardiac remodelling in DCM. (A) Comparison of the fasting blood glucose levels (mg/dL) in CON (black diamonds; *n* = 14/13/14 rats) and STZ rats (white diamonds; *n* = 15/14/19 rats) at 4, 8 and 12 weeks after STZ or vehicle injection. (B) BW (g) in CON (black diamonds; *n* = 14/13/14 rats) and STZ rats (white diamonds; *n* = 15/14/19 rats) at 4, 8 and 12 weeks after STZ or vehicle injection. Statistical analysis was performed with a two‐way anova followed by a post hoc Tukey's multiple comparison test. Statistically significant differences between CON and STZ rats of the same age are indicated as ***, *p* < 0.001. Statistically significant differences between CON‐4 weeks and CON‐8 weeks or between STZ‐4 weeks and STZ‐8 weeks are indicated as ^^^^, *p* < 0.01; ^^^^^, *p* < 0.001. Statistically significant differences CON‐4 weeks and CON‐12 weeks or between STZ‐4 weeks and STZ‐12 weeks are indicated as ^&^, *p* < 0.05 and ^&&&^, *p* < 0.001. (C) HW (g) in CON (black diamonds; *n* = 14/13/14 rats) and STZ rats (white diamonds; *n* = 15/14/19 rats) at 4, 8 and 12 weeks after STZ or vehicle injection. (D) mRNA expression of ANF normalized to GAPDH measured in CON (black diamonds; *n* = 9/9/9 rats) and STZ rats (white diamonds; *n* = 9/10/9 rats) at 4, 8 and 12 weeks after STZ or vehicle injection. Two‐way anova analysis showed no interaction between STZ treatment and the time; however, STZ treatment alone had a statistically significant effect on HW and ANF expression (*p* < 0.0001). All data represent the mean ± S.E.M. HW, heart weight; ANF, atrial natriuretic factor; GAPDH, Glyceraldehyde 3‐phosphate dehydrogenase.

In order to delineate the alterations affecting cAMP effectors and the major actors of the cardiac ECC, we used a T1D‐induced DCM model, which we characterized in detail in a previous study.^37^ T1D was induced by STZ injection in 5‐week‐old rats (Figure S1), and glycaemia was measured in both vehicle‐treated (CON) and STZ‐treated (STZ) rats at 4, 8 and 12 weeks after injection. FBG levels were increased by ~4.4‐fold in STZ‐treated rats compared with their age‐matched CON (Figure 1A). Anatomical data of control and diabetic rats monitored at 4, 8 and 12 weeks following injection indicate that all STZ‐treated rats had a significantly lower BW (Figure 1B) as well as lungs, liver and kidneys weights compared with their age‐matched CON (Table 1). Moreover, a significant decrease in the heart weight (HW) by ~50% was noted in all STZ‐treated rats compared with their age‐matched CON (Figure 1C and Table 1) along with increases in the expression of ANF (Figure 1D), a biomarker gene for DCM. These data attest a structural remodelling in the diabetic hearts.

**TABLE 1:** Anatomical data of control (CON) and diabetic (STZ) rats at 4, 8 and 12 weeks after injection of either streptozotocin or vehicle.

	Week 4		Week 8		Week 12	
	CON (*n* = 14)	STZ (*n* = 15)	CON (*n* = 13)	STZ (*n* = 14)	CON (*n* = 14)	STZ (*n* = 19)
BW (g)	284 ± 6	100 ± 8***	358 ± 9^^^^^	135 ± 9***	367 ± 13^&&&^	140 ± 7***^;&^
HW (g)	0.88 ± 0.03	0.40 ± 0.03	1.04 ± 0.04	0.52 ± 0.02	1.06 ± 0.04	0.57 ± 0.02
HW/BW (mg/g)	3.1 ± 0.1	4.1 ± 0.2	2.9 ± 0.1	3.9 ± 0.1	2.9 ± 0.1	4.2 ± 0.2
Lungs (g)	1.52 ± 0.06	0.76 ± 0.04	1.56 ± 0.06	1.02 ± 0.06	1.84 ± 0.10	1.09 ± 0.05
Lungs/BW (mg/g)	5.34 ± 0.16	7.91 ± 0.42	4.36 ± 0.16	7.79 ± 0.47	5.06 ± 0.26	8.15 ± 0.58
Kidneys (g)	1.96 ± 0.06	1.31 ± 0.09	2.43 ± 0.09	1.74 ± 0.09	2.32 ± 0.09	1.81 ± 0.07
Kidney/BW (mg/g)	6.89 ± 0.14	13.29 ± 0.34	6.77 ± 0.17	13.07 ± 0.28	6.34 ± 0.15	13.43 ± 0.78
Liver (g)	9.98 ± 0.61	4.47 ± 0.33	11.17 ± 0.66	6.62 ± 0.46	11.19 ± 0.45	7.11 ± 0.33
Liver/BW (mg/g)	35.05 ± 1.99	45.52 ± 1.91*	31.02 ± 1.21	49.57 ± 2.05***	30.77 ± 1.22	52.54 ± 3.29***

*Note*: All data are expressed as mean ± S.E.M. A two‐way anova test was performed to study the effect of STZ treatment, time and their interaction on the different outcomes. The interaction was only significant for the BW and Liver/BW; hence, two‐way anova was followed by post hoc Tukey's multiple comparison test. Statistically significant differences between CON and STZ rats of the same age are indicated as *, *p* < 0.05; ***, *p* < 0.001. Statistically significant differences between CON‐4 weeks and CON‐8 weeks are indicated as ^^^, *p* < 0.001. Statistically significant differences between CON‐4 weeks and CON‐12 weeks or between STZ‐4 weeks and STZ‐12 weeks are indicated as ^&^, *p* < 0.05; ^&&&^, *p* < 0.001. Two‐way anova showed no significant interaction between STZ treatment and time for the HW, HW/BW, Lungs, Lungs/BW, Kidneys, Kidneys/BW and liver, while the effect of STZ treatment alone had the same statistically significant effect on all these outcomes (*p* < 0.0001) across time.

Abbreviations: BW, body weight; HW, heart weight.

### 3.2 Expression of Epac1/2 isoforms in control and diabetic hearts

**FIGURE 2:**
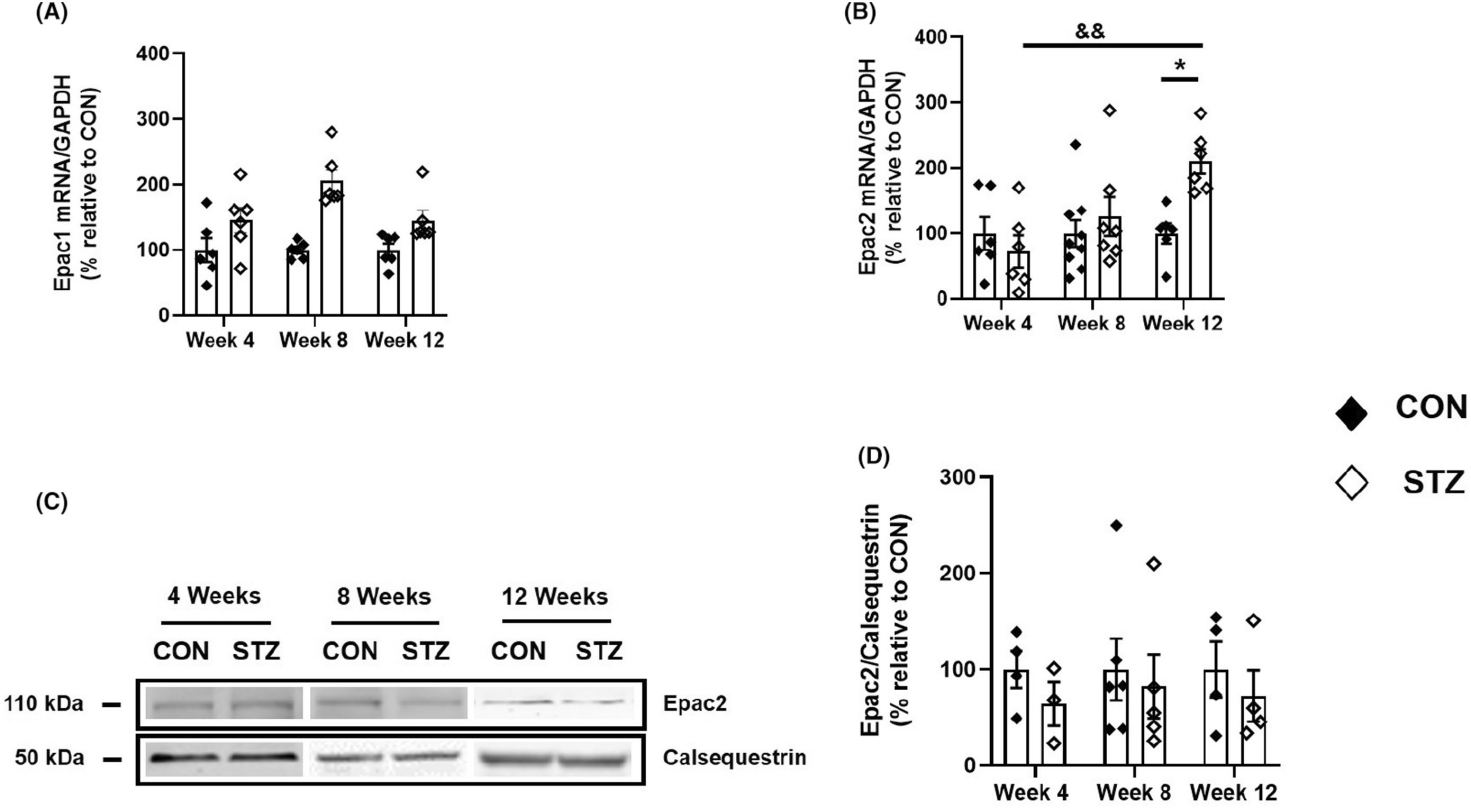
Expression of Epac1 and Epac2 isoforms in hearts from control and diabetic rats at 4, 8 and 12 weeks. (A). Epac1 mRNA expression in CON (black diamonds; *n* = 6/6/6 rats) and STZ rats (white diamonds; *n* = 6/6/6 rats) at 4, 8 and 12 weeks after STZ or vehicle injection. (B) Epac2 mRNA expression in CON (black diamonds; CON: 6/9/6 rats) and STZ rats (white diamonds; STZ: 6/7/6 rats) at 4, 8 and 12 weeks after STZ or vehicle injection. mRNA expression of both isoforms was normalized to GAPDH in both STZ and aged‐matched CON rats. Two‐way anova test showed no significant interaction between STZ treatment and time; however, STZ treatment alone had a statistically significant effect on Epac1 mRNA expression (*p* < 0.0001). A two‐way anova followed by a post hoc Tukey's multiple comparison test was performed to assess the changes in the expression of Epac2 mRNA. Statistically significant differences between CON and STZ rats of the same age are indicated as *, *p* < 0.05. Statistically significant differences between STZ‐4 weeks and STZ‐12 weeks are indicated as ^&&^, *p* < 0.01. Equal amounts of cardiac proteins from control (CON) and diabetic rats (STZ) were separated on SDS/PAGE and revealed with Epac2‐specific antibody. Calsequestrin was used as a loading control. (C) Shown is a representative blot for Epac2 in CON and STZ at 4, 8 and 12 weeks. (D) Quantification of all data obtained in several immunoblots from hearts of CON (black diamonds; *n* = 4/6/4) and STZ (white diamonds; *n* = 3/5/4) at 4, 8 and 12 weeks, respectively, and represented as mean ± S.E.M. Statistical analysis was performed with two‐way anova tests that showed no significant differences in Epac2 mRNA expression between CON and STZ rats.

To determine whether DCM impacts cAMP downstream signalling, we first measured mRNA expression of Epac1 and Epac2 in hearts from control and diabetic rats at 4, 8 and 12 weeks following either vehicle or STZ injection. Each cardiac sample was then normalized to GAPDH levels, which were similar between STZ‐treated rats and their age‐matched CON (Figure S2). Statistical analysis showed no significant interaction between STZ treatment and the time on the mRNA expression of Epac1; however, STZ treatment induced significant increases in its expression, similarly across time (*p* < 0.001; Figure 2A). In contrast, the upregulation in Epac2 transcripts by ~2‐fold was not apparent in STZ‐treated rats compared with their age‐matched CON until Week 12 (*p* < 0.05; Figure 2B). We then determined whether the strong increase in Epac2 mRNA observed in the diabetic hearts translates into similar changes in protein expression. Equal amounts of proteins prepared from heart extracts from CON or STZ rats were separated on SDS/PAGE, and Epac2 protein was detected by western blotting using Epac2 selective antibody. A single band migrating at approximately 110 kDa was detected in rat hearts for Epac2 (Figure 2C; Figure S3) and its expression did not change in diabetic rats compared with their age‐matched CON regardless of the disease stage (Figure 2D).

### 3.3 Expression of PKA subunits and CaMKII in control and diabetic hearts

**FIGURE 3:**
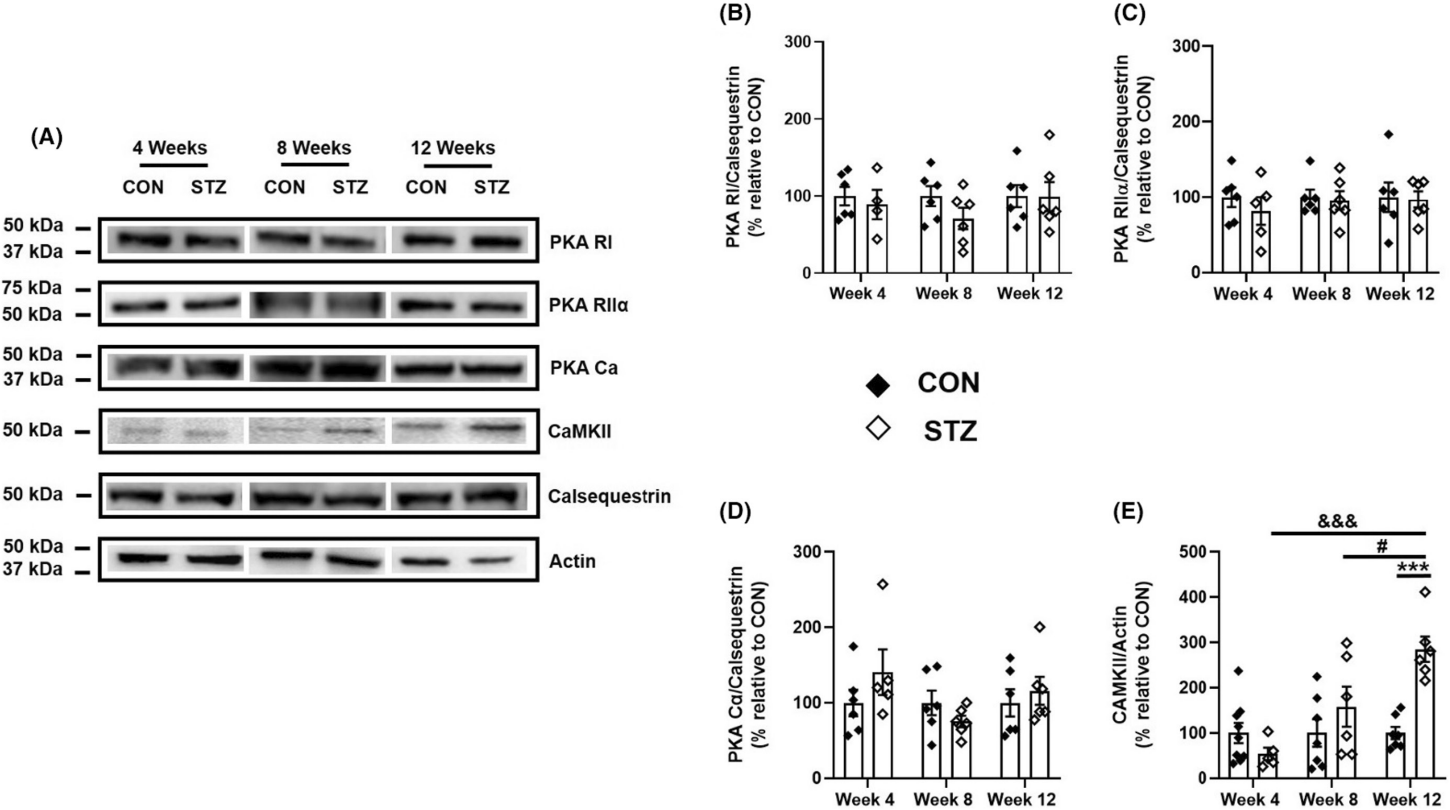
Expression of PKA subunits and CaMKII in hearts from control and diabetic rats at 4, 8, and 12 weeks. Equal amounts of cardiac proteins from control (CON) and diabetic rats (STZ) were separated on SDS/PAGE and revealed with PKA RI‐, PKA RIIα‐, PKA Cα‐, CaMKII‐specific antibodies. Calsequestrin and actin were used as loading controls. (A) Representative blots for PKA RI, PKA RIIα, PKA Cα, CaMKII, calsequestrin and actin in CON and STZ at 4, 8 and 12 weeks are shown. (B–E) Quantification of all data obtained in several immunoblots from hearts of CON (black diamonds) and STZ (white diamonds) and represented as mean ± S.E.M. In (B–D), two‐way anova showed no significant interaction between STZ treatment and time. Additionally, neither STZ treatment alone nor the time had any statistically significant effect on the expression of PKA RI (CON: *n* = 6/6/6; STZ: *n* = 4/6/6 at 4, 8 and 12 weeks, respectively), PKA RIIα (CON: *n* = 6/6/6; STZ: *n* = 5/6/6 at 4, 8 and 12 weeks, respectively) and PKA Cα (CON: *n* = 6/6/6; STZ: *n* = 5/6/6 at 4, 8 and 12 weeks, respectively) between CON and STZ rats. In (E), two‐way anova followed by a post hoc Tukey's multiple comparison test was performed to assess the changes in the expression of CaMKII (CON: *n* = 9/7/7; STZ: *n* = 5/6/6 at 4, 8 and 12 weeks, respectively) that were dependent on both the time and STZ treatment as well as on the interaction of both factors. Statistically significant differences between CON and STZ rats of the same age are indicated as ***, *p* < 0.001. Statistically significant differences between STZ‐4 weeks and STZ‐12 weeks are indicated as ^&&&^, *p* < 0.001. Statistically significant differences between STZ‐8 weeks and STZ‐12 weeks are indicated as ^#^, *p* < 0.05.

We then examined whether the expression of PKA, another cAMP effector playing a pivotal role in the regulation of cardiac contraction,^13^ is altered in DCM. Different PKA subunits were investigated in hearts from control and diabetic rats at 4, 8 and 12 weeks following either vehicle or STZ injection. We detected a single band migrating at approximately 49 kDa for PKA RI (Figure 3A; Figure S4), 51 kDa for PKA RIIα (Figure 3A; Figure S5) and 40 kDa for PKA Cα (Figure 3A; Figure S6), respectively, in both control and diabetic hearts.

CaMKII is another cardiac kinase exhibiting a key role in tuning ECC.^15^ One band migrating at approximately 50 kDa was identified for CaMKII in rat hearts (Figure 3A; Figure S7).

Each cardiac sample was then normalized to calsequestrin and/or actin, since the expression of either protein was similar between STZ‐treated rats and their age‐matched CON (Figure 3A). As shown in Figure 3B,C,D, no statistical difference was observed in the cardiac expression of PKA RI, PKA RIIα and PKA Cα between control and diabetic rats, regardless of the disease stage. In contrast, CaMKII expression was strongly increased in diabetic hearts at 12 weeks compared with their age‐matched controls (*p* < 0.001; Figure 3E).

### 3.4 Expression of the major actors of cardiac ECC in hearts from control and diabetic rats

**FIGURE 4:**
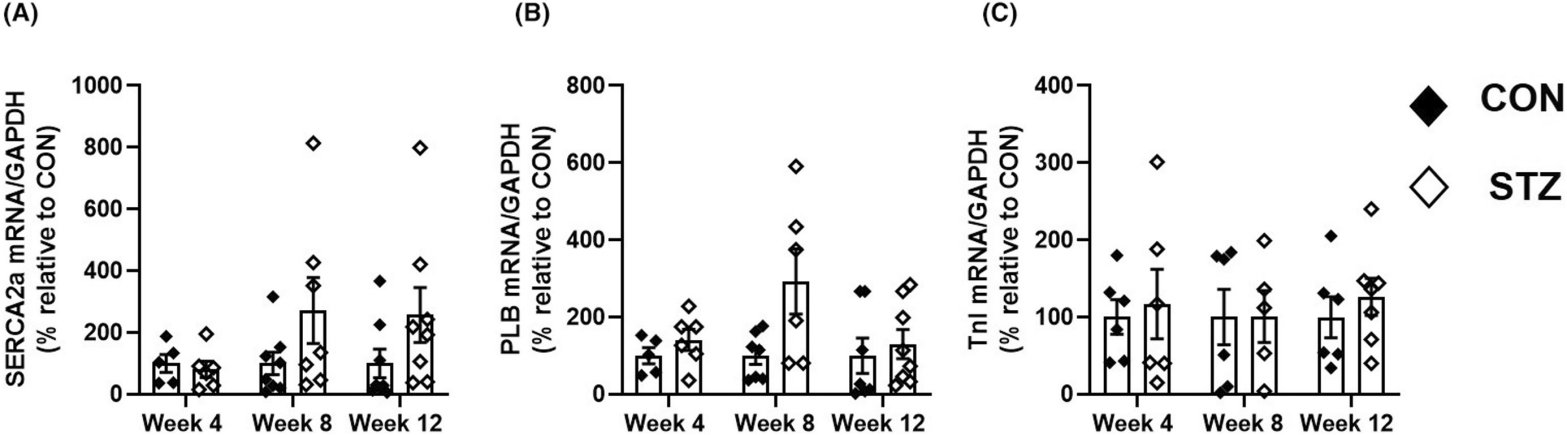
Expression of the major actors of the cardiac ECC in hearts from control and diabetic rats at 4, 8, and 12 weeks. Total RNA was extracted from hearts of 5 to 8 control (CON, black diamonds) and diabetic rats (STZ, white diamonds) at each time point and analysed by Real‐time PCR for SERCA2a (A), PLB (B) and TnI (C). mRNA expression was normalized to GAPDH in both STZ and aged‐matched CON rats. Data represent the mean ± S.E.M. Statistical analysis was performed with two‐way anova that showed no significant interaction between STZ treatment and time for all proteins. However, STZ treatment had the same statistically significant effect on PLB (*p* < 0.05) across time.

In a next series of experiments, we examined the variations in the mRNA expression of the major actors of the cardiac ECC (SERCA2a, PLB and TnI) in hearts from control and diabetic rats at 4, 8 and 12 weeks. Each cardiac sample was then normalized to GAPDH levels which were similar between STZ‐treated rats and their age‐matched CON (Figure S2). Statistical analysis showed no significant interaction between STZ treatment and the time on the mRNA expression of SERCA2a, PLB and TnI (Figure 4). However, STZ treatment induced a significant increase in PLB mRNA expression similarly across time (*p* < 0.05) whereas SERCA2a and TnI mRNA expression did not change in diabetic compared with control rats regardless of the disease stage.

**FIGURE 5:**
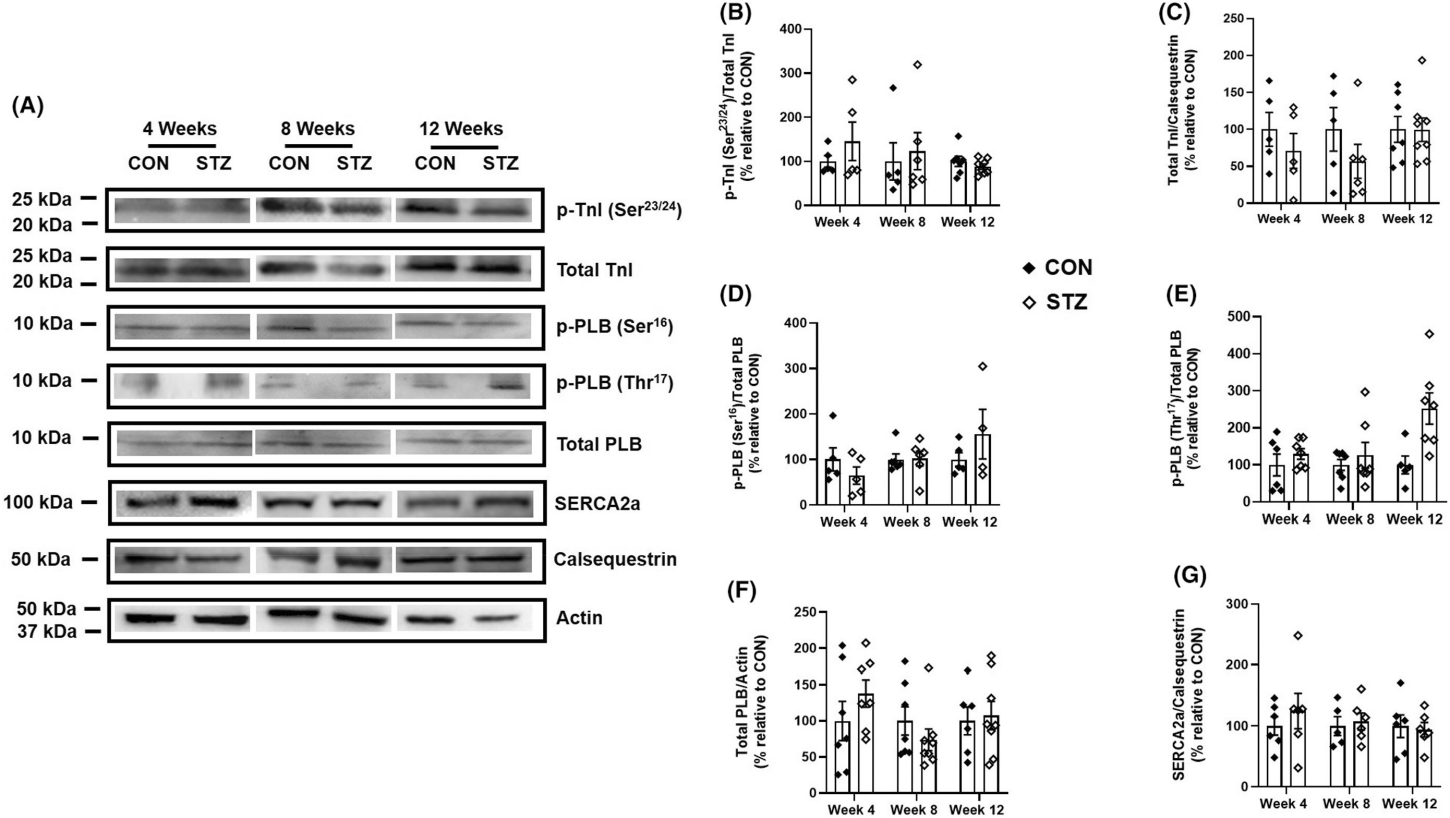
Expression of TnI, PLB and SERCA2a proteins in hearts from control and diabetic rats at 4, 8, and 12 weeks. Equal amounts of cardiac proteins from control (CON) and diabetic rats (STZ) were separated on SDS/PAGE and revealed with p‐TnI (Ser^23/24^), TnI, p‐PLB (Ser^16^), p‐PLB (Thr^17^), PLB and SERCA2a‐specific antibodies. Calsequestrin and actin were used as loading controls. (A), Representative blots for p‐TnI (Ser^23/24^), TnI, p‐PLB (Ser^16^), p‐PLB (Thr^17^), PLB, SERCA2a, calsequestrin and actin in CON and STZ at 4, 8 and 12 weeks are shown. (B–G) Quantification of all data obtained in several immunoblots from hearts of 4 to 8 CON (black diamonds) and STZ (white diamonds) and represented as mean ± S.E.M. Statistical analysis was performed with two‐way anova tests that showed no significant interaction between STZ treatment and time for all proteins. However, STZ treatment alone had the same statistically significant effect on p‐PLB (Thr^17^)/Total PLB (*p* < 0.01) across time.

We next determined whether the mRNA expression pattern of SERCA2a, PLB and TnI in DCM (Figure 4) translates into a similar protein expression profile. A single band migrating at approximately 24 kDa for total TnI (Figure 5A; Figure S9), 7 kDa for total PLB (Figur 5A; Figure S12) and 100 kDa for SERCA2a (Figure 5A; Figure S13) was detected in both control and diabetic hearts. Expression of total TnI, PLB and SERCA2a was similar in CON and STZ‐treated rats at 4, 8 and 12 weeks (Figure 5A–C,F,G).

In healthy myocardium, PLB is phosphorylated at Ser^16^ by PKA and at Thr^17^ by CaMKII.^38^, ^39^ Phosphorylation of TnI by PKA and PKC occurs at both Ser^23^ and Ser^24^ and leads to the depression of myofilament Ca^2+^ sensitivity.^40^, ^41^, ^42^, ^43^, ^44^, ^45^, ^46^ We tested whether these phosphorylation events were altered in the diabetic myocardium. Proteins were extracted from hearts of CON and STZ‐treated rats at 4, 8 and 12 weeks, and the expression of p‐TnI (Ser^23^/^24^), p‐PLB (Ser^16^) and p‐PLB (Thr^17^) was determined by western blotting as shown in Figure 5A. No significant differences were noted in the p‐TnI (Ser^23^/^24^)/Total TnI and p‐PLB (Ser^16^)/Total PLB ratios (Figure 5B–D; Figures S8 and S10) between control and diabetic rats regardless of the disease stage. Interestingly, the phosphorylation of PLB (Thr^17^) normalized to total PLB was increased by diabetes across time (*p* < 0.01; Figures 5E; Figure S11).

## 4 DISCUSSION

The major goal of the present study was to determine whether the differential and time‐specific changes that we have previously reported in cardiac β‐AR/cAMP/PDEs signalling^37^ are associated with modifications of the downstream components of cAMP pathway and in the main players of cardiac ECC in a T1D‐induced DCM model. Our results show that T1D induces time and isoform‐specific modifications in the expression of cAMP effectors, particularly Epac, CaMKII and the major Ca^2+^ handling proteins. Indeed, an early increase in Epac1 transcripts was noted in diabetic hearts at week 4, followed by increases in Epac2 mRNA at Week 12. However, Epac2 protein levels were unchanged. Expression of PKA subunits (RI, RIIα and Cα) remained unchanged with the progression of DCM, whereas CaMKII increased at Week 12. Changes in the various Ca^2+^ handling proteins involved a specific regulation at both the transcriptional and translational levels as the disease progressed. Indeed, PLB transcripts, but not protein levels, were upregulated in diabetic hearts with no changes observed in SERCA2a and TnI gene expression irrespective of the disease evolution. Moreover, PLB phosphorylation at threonine‐17 was increased in DCM, whereas phosphorylation of both PLB at serine‐16 and TnI at serine‐23/24 remained unchanged.

**FIGURE 6:**
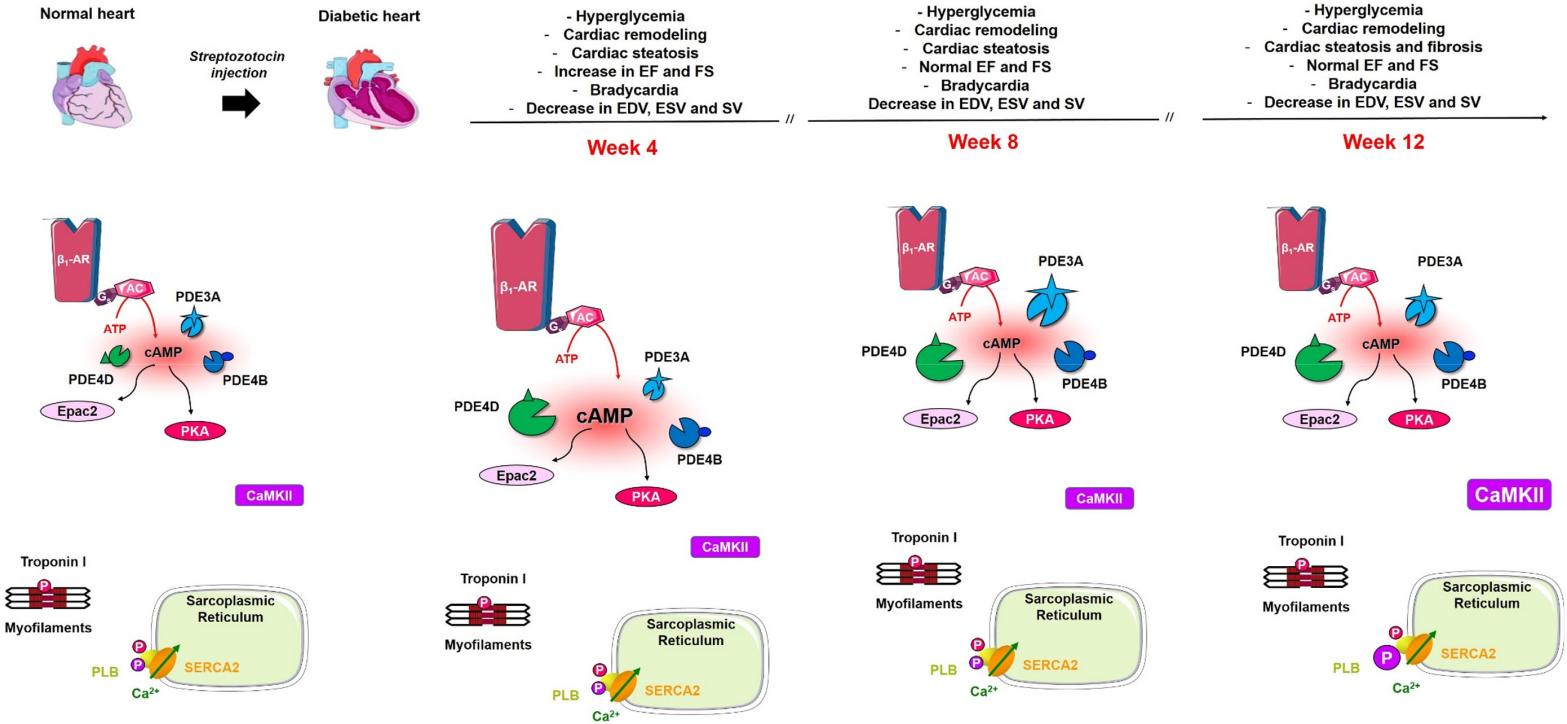
Summary of cAMP and Ca^2+^ signalling pathways remodelling during DCM. We have previously shown that 4 weeks after STZ injection, a modest increase in cAMP content, although not significant, resulted from a balance between β_1_‐AR receptors, PDE4B and PDE4D whose expression was upregulated in the diabetic heart.^37^ At Weeks 8 and 12, β_1_‐AR receptors and cAMP content were restored while the expression of PDE3A was increased.^37^ In the present study, Epac2 protein levels as well as the expression of PKA subunits (RI, RIIα and Cα) remained unchanged with the progression of DCM, whereas CaMKII increased at Week 12 in the diabetic myocardium. Moreover, no changes were observed in the protein levels of PLB, SERCA2a and TnI irrespective of the disease evolution. Importantly, PLB phosphorylation at threonine‐17 was increased in DCM, whereas phosphorylation of both PLB at serine‐16 and TnI at serine‐23/24 remained unchanged (see text for more details). EF, ejection fraction; FS, fraction shortening; EDV, end‐diastolic volume; ESV, end‐systolic volume; SV, stroke volume; β_1_‐AR, β_1_‐adrenergic receptor; AC, adenylyl cyclase; cAMP, cyclic adenosine 3′‐5′ monophosphate; PDE, phosphodiesterase; PKA, cAMP‐dependent protein kinase; Epac, guanine nucleotide‐exchange factor for the small GTPases Rap1 and Rap2; CaMKII, Ca^2+^/Calmodulin‐dependent kinase II; PLB, phospholamban; SERCA2a, sarcoplasmic reticulum Ca^2+^ pump.

The STZ‐induced T1D model used in the present study was recently characterized in detail. Consistent with our previous findings,^37^ sustained hyperglycaemia measured in STZ‐injected rats was associated with decreases in HW by ~50% and increases in both HW indexed to BW (~ +38%) and ANF expression, a cardiac remodelling marker of the foetal gene programme. Although not repeated in the present series, our previous data showed that STZ‐treated rats exhibited other known structural hallmarks of the diabetic heart as early as the 4th week, including a reduction of mean cardiac myocyte width and the emergence of cardiac steatosis, whereas we noted a relatively late onset of cardiac fibrosis on the 12th week following STZ injection (Figure 6). Furthermore, echocardiography pointed to the development of DCM‐associated cardiac remodelling, namely decreases in LV posterior and septal wall thicknesses, LV end‐systolic and end‐diastolic diameters, as well as a decrease in heart rate (HR) and LV volumes during the cardiac cycle at 4, 8 and 12 weeks after STZ injection. Both EF and FS were first increased in STZ‐treated rats at 4 weeks in line with concomitant upregulation of β_1_‐AR receptors and cAMP levels.^37^ However, as the disease progressed, cardiac function along with β_1_‐AR transcripts and total basal cAMP levels were normalized, whereas PDE3A protein expression increased at Week 8.^37^ Thus, in the present study, we assessed whether these changes affect cAMP effectors, particularly Epac1/2 and PKA. Our results show an increase in Epac1 mRNA expression in diabetic hearts at Week 4, which coincided with increases in β_1_‐AR/cAMP in the same rat model. However, Epac1 upregulation is maintained until Week 12 when elevation in Epac2 mRNA expression also emerges. Interestingly, alterations in Epac2 mRNA seen in diabetic rats were not reflected in similar changes of the protein expression regardless of the disease stage. In the myocardium from both control and diabetic adult rats, Epac1 protein expression was not detected with the antibody tested in this study. This could be related to developmental changes in gene expression of Epac isoforms, as some studies reported that Epac2, relative to Epac1, becomes dominant in the adult compared with foetal heart.^22^ Although the mechanisms were not explored, our results suggest that the genes encoding for Epac1 and Epac2 are subjected to time‐specific and differential regulation of their transcription and translation. Furthermore, one cannot exclude the fact that the twelve‐week timeline followed in this study to assess the molecular changes underlying the progression of DCM lies most probably within the pathophysiological window that precedes any alterations occurring in Epac2 protein levels. Moreover, potential specific changes in the expression of Epac1/2 proteins may occur in subcellular cardiac compartments that would require more precise investigations.^47^, ^48^ Indeed, Epac1 was shown previously to mediate the pro‐hypertrophic effects of β‐AR stimulation^49^ through either its recruitment to β_1_‐ARs via β‐arrestin2^47^ or its association with PDE4D3, RyR2, calcineurin and the extracellular signal‐regulated kinase 5 (ERK5) in a mAKAP (muscle A kinase‐anchoring protein)‐coordinated signal transduction complex at the nuclear envelope.^50^ Moreover, Epac2 has been suggested to induce SR Ca^2+^ leak and arrhythmia, an effect that seems to be mediated by β_1_‐AR pathway.^51^ Taken together, these alterations might participate in the progression of DCM. Interestingly, expression of PKA RI, PKA RIIα and PKA Cα was unchanged in DCM regardless of the disease stage, attesting differential regulation of PKA and Epac expression in DCM and hence distinctive implications of these two effectors in cAMP signalling in cardiac diseases. Our results on PKA subunits are consistent with previous studies that showed similar protein content of PKA RI, RII and C subunits despite a decrease in PKA activity in the heart of mice at 16 weeks after induction of T1D.^32^ However, when investigating specific changes affecting the C subunit of PKA in different cellular compartments, the protein levels were decreased in the cytosolic and myofilaments/nuclear fractions.^32^ This compartment‐specific loss of PKA, which was reflected by reduced phosphorylation of discrete substrates and was attributed to contractile impairment, points at the potential remodelling of cAMP/PKA pools in the diabetic myocardium. Although we did not measure PKA activity in this study, the unchanged PKA‐dependent phosphorylation of PLB and TnI that we report herein could speak for a preserved PKA activity in diabetic hearts at least in the vicinity of these two targets. Indeed, similar basal PKA activity was reported in the heart of rats at 6 weeks after induction of T1D.^52^ Nevertheless, controversial findings were also reported concerning both basal and stimulated cardiac PKA activity in STZ‐induced T1D, which might be due to species differences and the duration of diabetes.^33^, ^53^

CaMKII, a critical transducer of Ca^2+^ signalling, is a multifunctional protein kinase that phosphorylates a wide range of substrates and regulates numerous cellular functions, including ECC.^35^, ^54^ CaMKII has been proposed as a key contributor to the deleterious effects of chronic β‐AR activation in DCM, primarily by exacerbating RyR2‐mediated diastolic Ca^2+^ leak.^55^, ^56^ Therefore, we determined whether the expression of CaMKII is altered in the diabetic myocardium during the progression of DCM. A single immunoreactive band migrating at ~50 kDa was detected in rat hearts for CaMKII. CaMKII δ isoform is the predominant isoform in the heart, including in human myocardium whereas the ɣ subunit is only expressed at low levels in cardiomyocytes.^54^ Interestingly, 12 weeks after STZ injection, DCM was associated with increases in CaMKII protein levels and CaMKII‐dependent phosphorylation of PLB. The rate of PLB phosphorylation may be the result of a balance between an upregulation in the kinase activity, which has been reported in DCM through phosphorylation, oxidation and O‐GlcNAcylation in diabetic hearts from rodents and human patients^57^, ^58^, ^59^, ^60^ and changes in phosphatases activity.^61^, ^62^

In DM, the process of cardiac Ca^2+^ cycling is modified in both humans and animal models, contributing to impaired cardiac contraction and relaxation.^30^ We characterized herein the changes affecting some of the main actors of ECC/ Ca^2+^ proteins handling, SERCA2a, PLB and TnI. SERCA2a mediates SR Ca^2+^ reuptake and therefore is a key determinant of both cardiac relaxation and contraction. Its activity is dependent on its interaction with PLB, which binds and inhibits the Ca^2+^ pump. However, phosphorylation of PLB relieves this inhibition and induces a substantial increase in Ca^2+^ flux via SERCA2a. Previous studies in diabetic animals have reported a reduction in the protein and/or the activity of SERCA2a, causing a decrease in the Ca^2+^ reuptake rate and SR Ca^2+^ load, hence contributing to cardiac dysfunction.^30^, ^63^ Moreover, PLB levels were reported to be higher in DCM^64^ whereas expression of p‐PLB was decreased.^63^, ^65^ In the present study, we report similar transcripts and protein levels of SERCA2a in controls and diabetic hearts at 4, 8 and 12 weeks following either STZ or vehicle injection. Protein expression of PLB was unchanged despite an increase in PLB mRNA levels at the three time points. Furthermore, SERCA2a‐to‐PLB ratio was also similar in control and diabetic myocardium (data not shown). Importantly, a higher basal phosphorylation rate of PLB by CaMKII, but not PKA, was detected at Week 12. One would still argue that differences in p‐PLB at serine‐16 can be unmasked upon ß‐AR stimulation, a possibility that we perfectly acknowledge. However, in light of the reported data herein, one would think that the steady‐state hyperphosphorylation of PLB leads to an upregulation of SERCA2a activity and a consequent improved Ca^2+^ transportation. These results are at variance with observations that Ca^2+^ reuptake into the SR was impaired in 7‐week diabetic rats, although the expression of SERCA2a, PLB and p‐PLB remained intact.^53^ The molecular mechanisms underlying the decrease in SERCA2a activity are not completely clear, when no changes in PLB expression or phosphorylation are found. Yet, several studies reported multiple possible mechanisms causing the reduction in SERCA2a activity in DCM. For instance, impairment of relaxation observed in DCM was shown to be associated with a decrease in the activity of SERCA2a due either to the direct damage of its ATP‐binding site by oxygen‐derived free radicals^66^, ^67^ or to its downregulation through posttranslational modifications, including glycation, carbonylation and O‐GlcNAcylation.^31^, ^68^ Taken together, these findings imply that hyperglycaemia leads to delayed Ca^2+^ uptake into the SR, thus affecting the normal processes of cardiomyocyte relaxation‐contractility; whereas SERCA2a expression/activity, and the expression/phosphorylation its primary regulator PLB, may remain normal or even stronger to compensate for this abnormality. It is worth noting that although we did not measure Ca^2+^ transients, a limitation of our study, the modifications in PLB phosphorylation by CaMKII but not PKA in the diabetic myocardium suggest that specific subcellular cAMP/PKA and Ca^2+^/CaMKII pools might be preferentially impacted in DCM and would require further characterization.

Abnormalities in the contractile and regulatory proteins could be responsible for the mechanical defects in DCM. However, there have been diverging findings regarding the sensitivity of the myofilament to Ca^2+^ in DCM.^69^, ^70^, ^71^ Phosphorylation of cardiac TnI is associated with altered Ca^2+^‐force relationship in isolated muscle preparations. Therefore, we investigated whether DM affects TnI expression and phosphorylation rate. Our results show similar expression of TnI at the protein level and phosphorylation at PKA sites in control and diabetic hearts. These findings are aligned with previous data published by our group, showing that STZ‐treated rats exhibit normal EF and FS as well as unchanged basal cAMP levels at 8, and 12 weeks following diabetes induction^37^ (Figure 6).

To the best of our knowledge, this is the first study that delineates the intricate regulations of cAMP effectors and the major actors of ECC in the myocardium during the pathophysiological progression of T1D‐induced DCM. These novel results are to be considered with respect to the previously documented changes affecting β‐AR/cAMP/PDEs signalling in the same animal model^37^ although they present some limitations. Importantly, the differential and time‐specific changes in cardiac Epac1/2, PKA subunits, CaMKII and the phosphorylation status of PLB and TnI in diabetic myocardium that we delineate herein at the level of the whole heart, suggest potential modifications in the regulation of specific subcellular cAMP and Ca^2+^ pools, which would require further characterization for a better understanding of how cAMP and Ca^2+^ compartmentalization is remodelled in DCM. Moreover, more subtle and specific changes in the phosphorylation status of some ECC actors may be unmasked upon ß‐AR stimulation with respect to basal conditions. One should note as well the potential confounding effects when assessing gene and protein expression in the whole heart versus cardiac myocytes. Indeed, the heart is comprised of a syncytium of cardiac myocytes and surrounding nonmyocytes, the majority of which are cardiac fibroblasts. In response to stress, cardiac myocytes become hypertrophic and can change their electrical properties, whereas fibroblasts convert into ‘activated’ myofibroblasts, proliferate and enhance ECM deposition, which leads to cardiac fibrosis.^72^ The latter affects cardiac myocyte metabolism and performance and ultimately ventricular function.^73^ We have previously documented the emergence of fibrosis in the diabetic myocardium 12 weeks following STZ injection.^37^ Accumulating evidence now suggests that downstream cAMP effectors such as PKA and Epac as well as Ca^2+^ signalling including CaMKII modulate a variety of fundamental cellular processes involved in fibroblasts and fibrosis‐associated cardiac diseases.^74^, ^75^, ^76^

## 5 CONCLUSION

In light of the novel data we provide herein, we conclude that abnormalities affecting cAMP effectors, CaMKII and Ca^2+^ proteins handling in DCM are complex and involve differential and time‐specific regulations. Our results suggest that more subtle and fine functional changes may occur in specific compartments within the cell. Thus, a fine characterization of these specific defects affecting both cardiac cAMP and Ca^2+^ signalling is crucial for a better understanding of the pathophysiology of DCM, its progression and management through the identification of new therapeutic targets.

## AUTHOR CONTRIBUTIONS

**Victoria Chaoul:** Conceptualization (equal); formal analysis (equal); investigation (equal); methodology (equal); visualization (equal); writing – original draft (equal). **Rita Hanna:** Conceptualization (equal); formal analysis (equal); investigation (equal); methodology (equal); visualization (equal). **Pia Hachem:** Investigation (equal). **Magali Samia El Hayek:** Investigation (equal). **Wared Nour‐Eldine:** Investigation (equal). **Pamela Abou‐Khalil:** Investigation (equal). **Elias Abi‐Ramia:** Investigation (equal). **Grégoire Vandecasteele:** Conceptualization (supporting); funding acquisition (supporting); resources (supporting); writing – review and editing (supporting). **Aniella Abi‐Gerges:** Conceptualization (lead); data curation (lead); funding acquisition (lead); methodology (lead); project administration (lead); resources (lead); supervision (lead); validation (lead); writing – original draft (lead); writing – review and editing (lead).

## CONFLICT OF INTEREST STATEMENT

The authors declare that there are no conflicts of interest. Magali Samia EL HAYEK is currently an employee of Eli Lilly and Company.

## Supporting information


Appendix S1.

## Data Availability

Data are available upon request.
