# Supplementary material for: Differential changes in cyclic adenosine 3′‐5′ monophosphate (cAMP) effectors and major Ca2+ handling proteins during diabetic cardiomyopathy
Source: J Cell Mol Med. 2023 Mar 27;27(9):1277–89. doi: 10.1111/jcmm.17733 (PMC10148055; doi:10.1111/jcmm.17733)
Supplement: Supplementary file 1 — Appendix S1. [file JCMM-27-1277-s001.docx]

**Supplementary Material**

**Differential Changes in Cyclic Adenosine 3′-5′ Monophosphate (cAMP) Effectors And Major Ca^2+^ Handling Proteins During
 Diabetic Cardiomyopathy**

**Chaoul *et al*.**

**Supplementary Methods**

**Animal model**

**
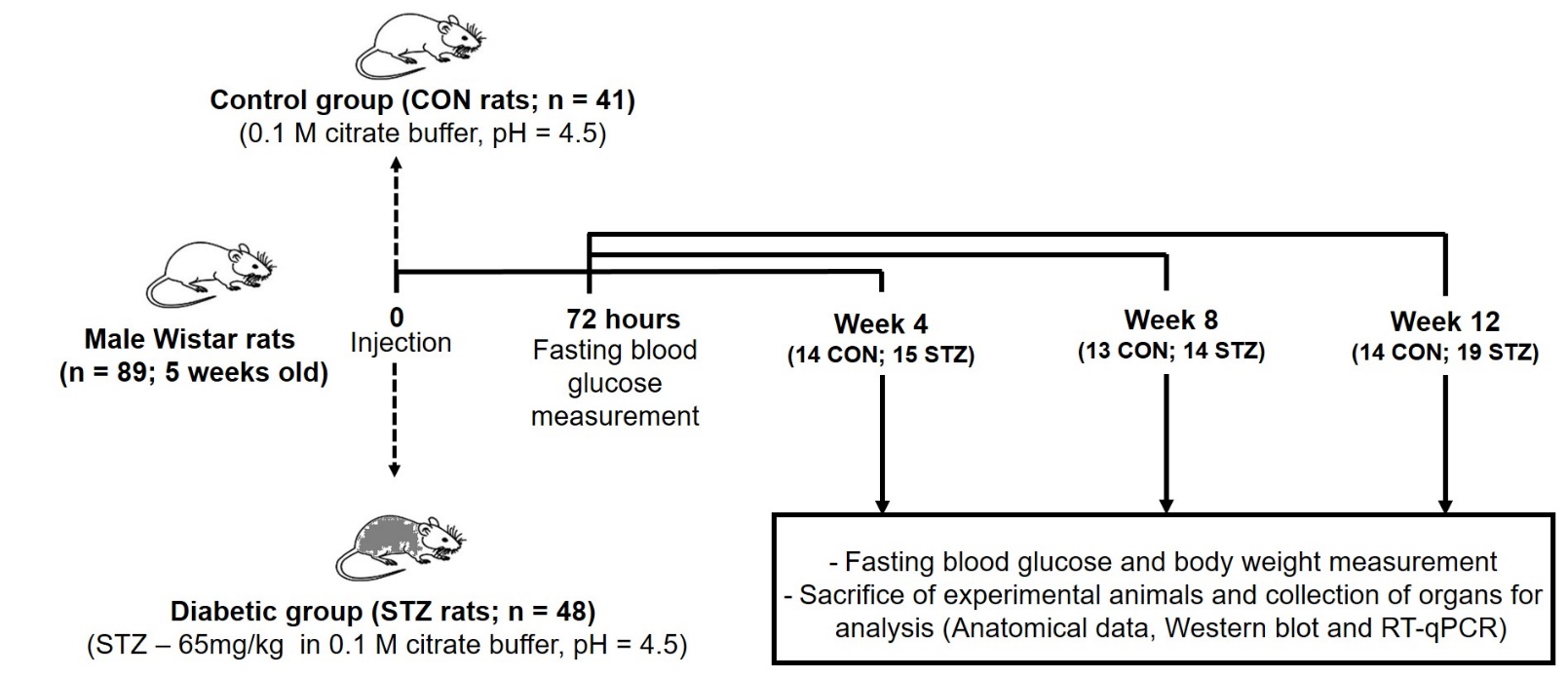
**

**Fig. S1. Experimental design of the study.** Five-week-old male Wistar rats (n = 89) were randomized into two groups. After 12 h fasting, one group received one intraperitoneal injection of streptozotocin (STZ, Sigma-Aldrich: 65 mg/kg in 0.1 M citrate buffer, pH = 4.5) to induce type 1 diabetes mellitus while the second age-matched control (CON) group was injected with vehicle (0.1 M citrate buffer, pH = 4.5). After 72 h, fasting blood glucose levels were measured and rats exhibiting blood glucose levels >200 mg/dL following STZ injection were considered diabetic. Four weeks after injection, glycemia and body weight of CON (n = 14) and STZ (n = 15) rats were evaluated. The animals were then sacrificed, and the organs were collected for further analysis. This procedure was repeated on the remaining rats at 8 (13 CON and 14 STZ) and 12 weeks (14 CON and 19 STZ) after STZ or vehicle injection.

**Real-time fluorescence quantitative PCR (RT-qPCR).**

**Table S1. Forward and reverse primers used for quantitative real time PCR.**

| ***Gene*** | ***Forward primer (5'-3')*** | ***Reverse primer (3'-5')*** |
| --- | --- | --- |
| **GAPDH** | TGC CAC TCA GAA GAC TGT GG | TTC AGC TCT GGG ATG ACC TT |
| **ANF** | ATC TGC CCT CTT GAA AAG CA | AAG CTG TTG CAG CCT AGT CC |
| **Epac1** | GAC GTC ACC ACT GCA AAC C | GCT GCC AGC TTG ATG AAC TT |
| **Epac2** | CCA CAC ATT TGG AAG GCA TA | GGG AAC AAA GGC AGA TCT CA |
| **PLB** | GCA GCT GAG CTC CCA GAC TT | TTT CCA TGA TGC CAG GAA GAC |
| **SERCA2a** | AGT GGC TGA TGG TGC TGA AA | GCA CCC GAA CAC CCT TAC AT |
| **TnI** | AGA TTG CGA AGC AGG AGA TG | AGC CCA TCC AAC ACC AAG |
|  |  |  |

**Supplementary Results**

**Fig. S2. Quantification cycle (Cq) of GAPDH in hearts from control and diabetic rats at 4, 8 and 12 weeks**.

GAPDH quantification cycle in CON (black diamonds; n = 9/9/9 rats) and STZ rats (white diamonds; n= 8/9/8 rats) at 4, 8 and 12 weeks after STZ or vehicle injection. Two-way ANOVA test showed no significant interaction between STZ treatment and time on GAPDH quantification cycle (*p* = 0.62).

**
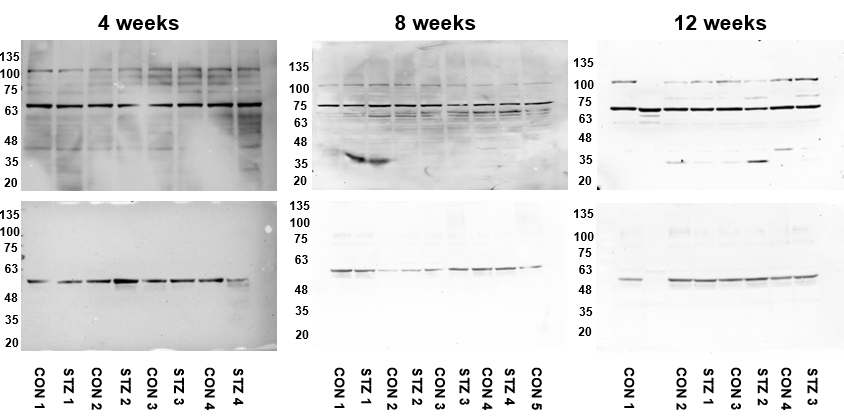
**

**CSQ 50 kDa**

**Epac2 110 kDa**

**Fig. S3. Original western blot for Epac2 in hearts from control and diabetic rats at 4, 8, and 12 weeks.** Equal amounts of cardiac proteins from control (CON) and diabetic rats (STZ) were separated on SDS/PAGE and revealed with Epac2 specific antibody. Calsequestrin (CSQ) was used as a loading control. Original blots for Epac2 and CSQ in CON (n = 4 to 5 rats) and STZ (n = 3 to 4 rats) at 4, 8 and 12 weeks are shown. A single band migrating at approximately 110 kDa was detected for Epac2 in both control and diabetic rat hearts. For CSQ, a single band migrating at 50 kDa was detected in rat hearts.


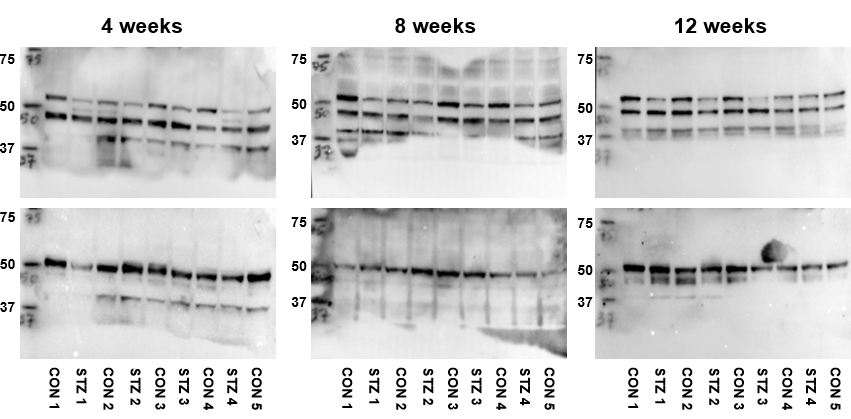


**CSQ 50 kDa**

**PKA RI 49 kDa**

**Fig. S4. Original western blot for PKA RI in hearts from control and diabetic rats at 4, 8, and 12 weeks.** Equal amounts of cardiac proteins from control (CON) and diabetic rats (STZ) were separated on SDS/PAGE and revealed with PKA RI specific antibody. Calsequestrin (CSQ) was used as a loading control. Original blots for PKA RI and CSQ in CON (n = 5 rats) and STZ (n = 4 rats) at 4, 8 and 12 weeks are shown. A single band migrating at approximately 49 kDa was detected for PKA RI in both control and diabetic rat hearts. For CSQ, a single band migrating at 50 kDa was detected in rat hearts.

**
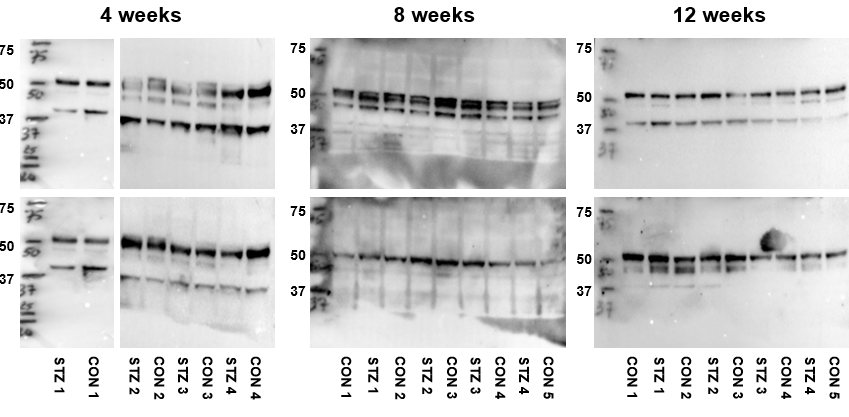
**

**CSQ 50 kDa**

**PKA RIIα 51 kDa**

**Fig. S5. Original western blot for PKA RII-α in hearts from control and diabetic rats at 4, 8, and 12 weeks.** Equal amounts of cardiac proteins from control (CON) and diabetic rats (STZ) were separated on SDS/PAGE and revealed with PKA RIIα specific antibody. Calsequestrin (CSQ) was used as a loading control. Original blots for PKA RIIα and CSQ in CON (n = 4 to 5 rats) and STZ (n = 4 rats) at 4, 8 and 12 weeks are shown. A single band migrating at approximately 51 kDa was detected for PKA RIIα in both control and diabetic rat hearts. For CSQ, a single band migrating at 50 kDa was detected in rat hearts.

**
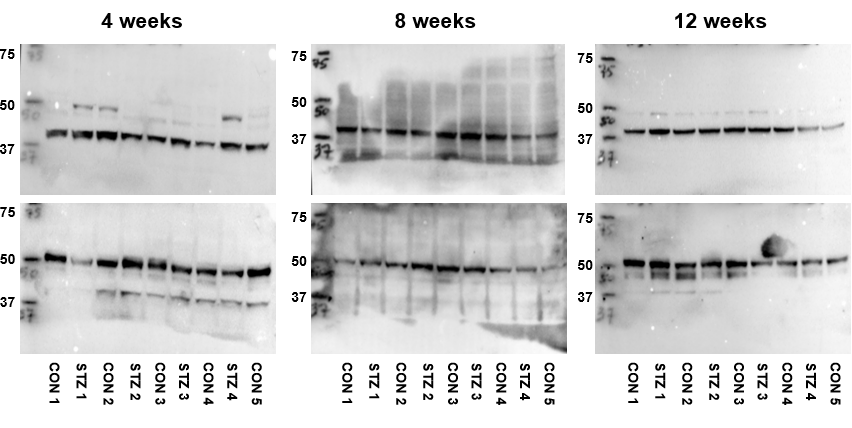
**

**CSQ 50 kDa**

**PKA Cα 40 kDa**

**Fig. S6. Original western blot for PKA Ca in hearts from control and diabetic rats at 4, 8, and 12 weeks.** Equal amounts of cardiac proteins from control (CON) and diabetic rats (STZ) were separated on SDS/PAGE and revealed with PKA Cα specific antibody. Calsequestrin (CSQ) was used as a loading control. Original blots for PKA Cα and CSQ in CON (n = 5 rats) and STZ (n = 4 rats) at 4, 8 and 12 weeks are shown. A single band migrating at approximately 40 kDa was detected for PKA Cα in both control and diabetic rat hearts. For CSQ, a single band migrating at 50 kDa was detected in rat hearts.

**
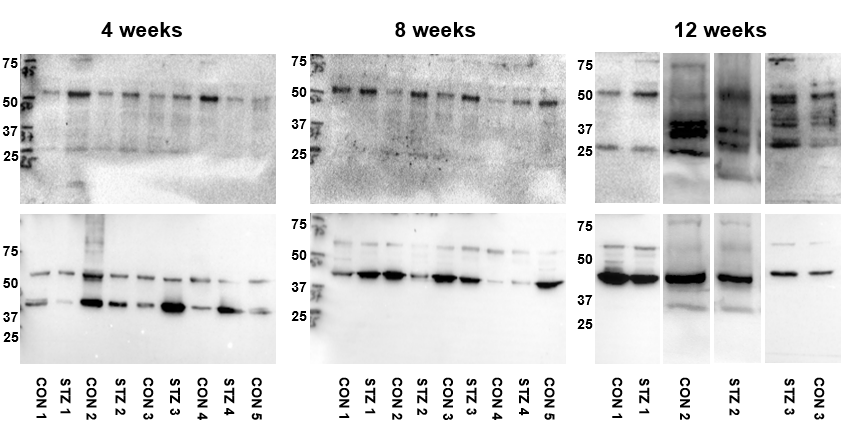
**

**Actin 42 kDa**

**CaMKII 50 kDa**

**Fig. S7. Original western blot for CaMKII in hearts from control and diabetic rats at 4, 8, and 12 weeks.** Equal amounts of cardiac proteins from control (CON) and diabetic rats (STZ) were separated on SDS/PAGE and revealed with CaMKII specific antibody. Actin was used as a loading control. Original blots for CaMKII and actin in CON (n = 3 to 5 rats) and STZ (n = 3 to 4 rats) at 4, 8 and 12 weeks are shown. A single band migrating at approximately 50 kDa was detected for CaMKII in both control and diabetic rat hearts. For actin, a single band migrating at approximately 42 kDa was detected in rat hearts.

**
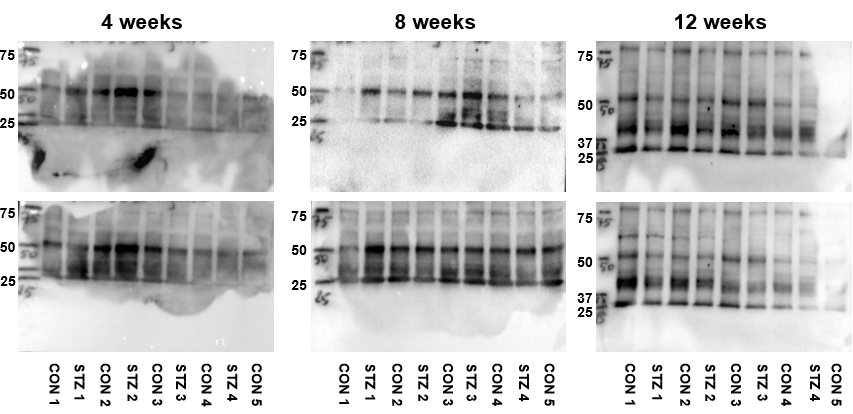
**

**Loading control**

**40 – 50 kDa**

**p-TnI (Ser^23/24^) 24 kDa**

**Fig. S8. Original western blot for p-TnI in hearts from control and diabetic rats at 4, 8, and 12 weeks.** Equal amounts of cardiac proteins from control (CON) and diabetic rats (STZ) were separated on SDS/PAGE and revealed with p-TnI (Ser^23/24^) specific antibody. Calsequestrin (CSQ) and actin were used as a loading control for 4/8 weeks and 12 weeks, respectively. Original blots for p-TnI, CSQ and actin in CON
(n = 5 rats) and STZ (n = 4 rats) at 4, 8 and 12 weeks are shown. A single band migrating at approximately 24 kDa was detected for p-TnI in both control and diabetic rat hearts. For CSQ, a single band migrating at approximately 50 kDa was detected in rat hearts whereas for actin, a single band was detected at 42 kDa.

**
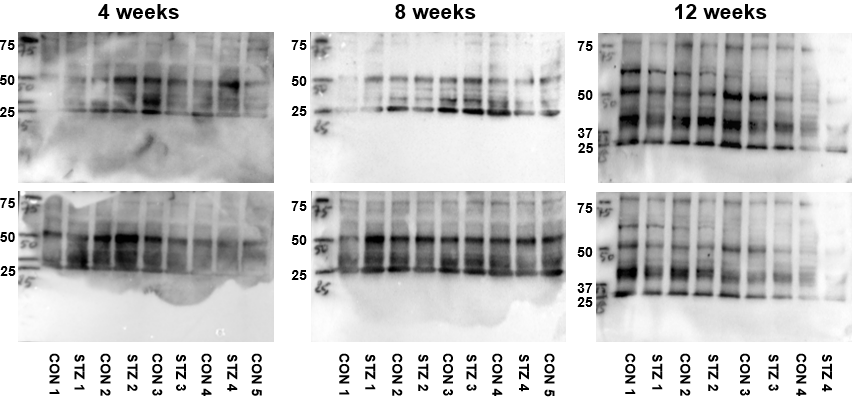
**

**Total TnI 24 kDa**

**Loading control**

**40 – 50 kDa**

**Fig. S9. Original western blot for Total TnI in hearts from control and diabetic rats at 4, 8, and 12 weeks.** Equal amounts of cardiac proteins from control (CON) and diabetic rats (STZ) were separated on SDS/PAGE and revealed with total TnI specific antibody. Calsequestrin (CSQ) and actin were used as a loading control 4/8 weeks and 12 weeks, respectively. Original blots for total TnI, CSQ and actin in CON (n = 4 to 5 rats) and STZ (n = 4 rats) at 4, 8 and 12 weeks are shown. A single band migrating at approximately 24 kDa was detected for total TnI in both control and diabetic rat hearts. For CSQ, a single band migrating at approximately 50 kDa was detected in rat hearts whereas for actin, a single band was detected at 42 kDa.

**
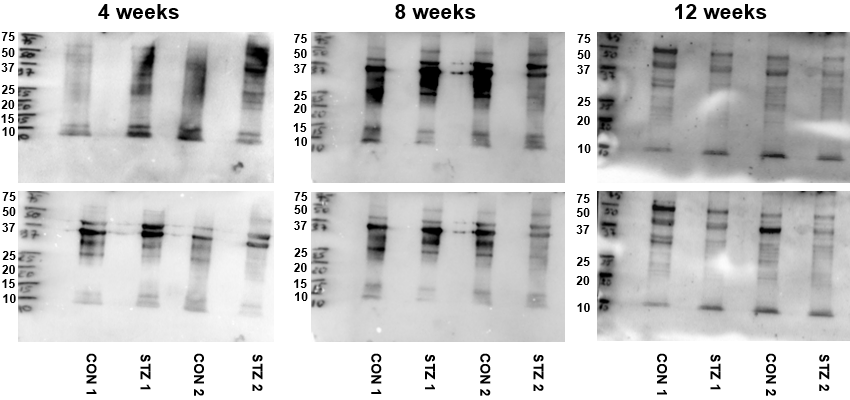
**

**Actin 42 kDa**

**p-PLB (Ser^16^) 7 kDa**

**Fig. S10. Original western blot for pPLB-Ser16 in hearts from control and diabetic rats at 4, 8, and 12 weeks.** Equal amounts of cardiac proteins from control (CON) and diabetic rats (STZ) were separated on SDS/PAGE and revealed with p-PLB (Ser^16^) specific antibody. Actin was used as a loading control. Original blots for p-PLB (Ser^16^) and actin in CON (n = 2 rats) and STZ (n = 2 rats) at 4, 8 and 12 weeks are shown. A single band migrating at approximately 7 kDa was detected for p-PLB (Ser^16^) in both control and diabetic rat hearts. For actin, a single band migrating at approximately 42 kDa was detected in rat hearts.

**
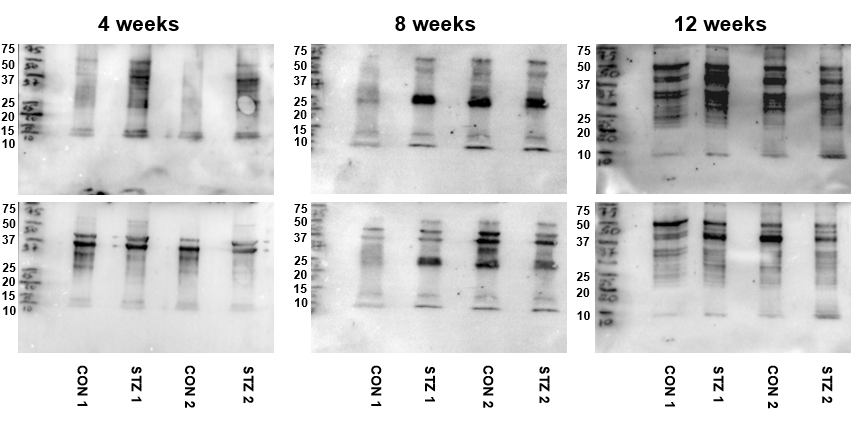
**

**Actin 42 kDa**

**p-PLB (Thr^17^) 7 kDa**

**Fig. S11. Original western blot for pPLB-Thr17 in hearts from control and diabetic rats at 4, 8, and 12 weeks.** Equal amounts of cardiac proteins from control (CON) and diabetic rats (STZ) were separated on SDS/PAGE and revealed with p-PLB (Thr^17^) specific antibody. Actin was used as a loading control. Original blots for p-PLB (Thr^17^) and actin in CON (n = 2 rats) and STZ (n = 2 rats) at 4, 8 and 12 weeks are shown. A single band migrating at approximately 7 kDa was detected for p-PLB (Thr^17^) in both control and diabetic rat hearts. For Actin, a single band migrating at approximately 42 kDa was detected in rat hearts.

**
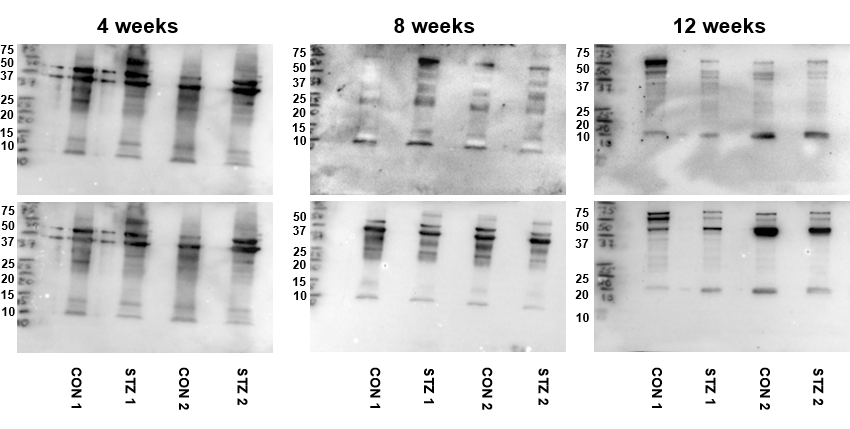
**

**Actin 42 kDa**

**Total PLB 7 kDa**

**Fig. S12. Original western blot for Total PLB in hearts from control and diabetic rats at 4, 8, and 12 weeks.** Equal amounts of cardiac proteins from control (CON) and diabetic rats (STZ) were separated on SDS/PAGE and revealed with total PLB specific antibody. Actin was used as a loading control. Original blots for total PLB and actin in CON (n = 2 rats) and STZ (n = 2 rats) at 4, 8 and 12 weeks are shown. A single band migrating at approximately 7 kDa was detected for total PLB in both control and diabetic rat hearts. For actin, a single band migrating at approximately 42 kDa was detected in rat hearts.

**
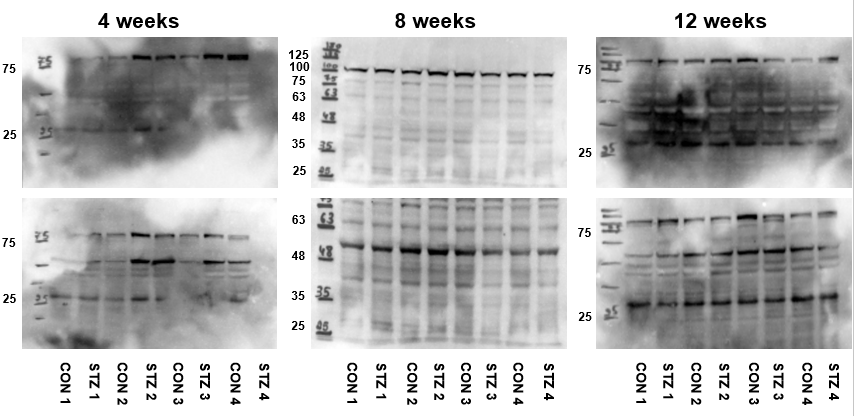
**

**CSQ 50 kDa**

**SERCA2a 100 kDa**

**Fig. S13. Original western blot for SERCA2a in hearts from control and diabetic rats at 4, 8, and 12 weeks.** Equal amounts of cardiac proteins from control (CON) and diabetic rats (STZ) were separated on SDS/PAGE and revealed with SERCA2a specific antibody. Calsequestrin (CSQ) was used as a loading control. Original blots for SERCA2a and CSQ in CON (n = 4 rats) and STZ (n = 4 rats) at 4, 8 and 12 weeks are shown. A single band migrating at approximately 100 kDa was detected for SERCA2a in both control and diabetic rat hearts. For CSQ, a single band migrating at 50 kDa was detected in rat hearts.
